# Jozimine A_2_, a Dimeric Naphthylisoquinoline (NIQ) Alkaloid, Shows In Vitro Cytotoxic Effects against Leukemia Cells through NF-κB Inhibition

**DOI:** 10.3390/ijms25063087

**Published:** 2024-03-07

**Authors:** Roxana Damiescu, Rümeysa Yücer, Sabine M. Klauck, Gerhard Bringmann, Thomas Efferth, Mona Dawood

**Affiliations:** 1 Department of Pharmaceutical Biology, Institute of Pharmaceutical and Biomedical Sciences, Johannes Gutenberg University, Staudinger Weg 5, 55128 Mainz, Germanyryuecer@students.uni-mainz.de (R.Y.); efferth@uni-mainz.de (T.E.); 2 Division of Cancer Genome Research, German Cancer Research Center (DKFZ) Heidelberg, National Center for Tumor Diseases (NCT), NCT Heidelberg, a Partnership between DKFZ and University Hospital, 69120 Heidelberg, Germany; 3 Institute of Organic Chemistry, University of Würzburg, Am Hubland, 97074 Würzburg, Germany; bringman@chemie.uni-wuerzburg.de

**Keywords:** autophagy, apoptosis, leukemia cells, jozimine A_2_, natural products, transcriptomics

## Abstract

Naphthylisoquinoline (NIQ) alkaloids are rising as a promising class of secondary metabolites with pharmaceutical potential. NF-κB has already been recognized as a significant modulator of cancer proliferation and drug resistance. We have previously reported the mechanisms behind the cytotoxic effect of dioncophylline A, an NIQ monomer, in leukemia cells. In the current study, we have investigated the cytotoxic effect of jozimine A_2_, an NIQ dimer, on leukemia cells in comparison to a second, structurally unsymmetric dimer, michellamine B. To this end, molecular docking was applied to predict the binding affinity of the dimers towards NF-κB, which was then validated through microscale thermophoresis. Next, cytotoxicity assays were performed on CCRF-CEM cells and multidrug-resistant CEM/ADR5000 cells following treatment. Transcriptome analysis uncovered the molecular networks affected by jozimine A_2_ and identified the cell cycle as one of the major affected processes. Cell death modes were evaluated through flow cytometry, while angiogenesis was measured with the endothelial cell tube formation assay on human umbilical vein endothelial cells (HUVECs). The results indicated that jozimine A_2_ bound to NF-κB, inhibited its activity and prevented its translocation to the nucleus. In addition, jozimine A_2_ induced cell death through apoptosis and prevented angiogenesis. Our study describes the cytotoxic effect of jozimine A_2_ on leukemia cells and explains the interactions with the NF-κB signaling pathway and the anticancer activity.

## 1. Introduction

A promising class of bioactive natural products are the naphthylisoquinoline (NIQ) alkaloids, such as dioncophylline A, isolated from tropical lianas of the Dioncophyllaceae and Ancistrocladaceae families [1,2]. We have recently shown that dioncophylline A and related NIQ alkaloids exhibit strong cytotoxicity in leukemia cells, mediated by NF-κB inhibition, angiogenesis suppression, G2/M cell cycle arrest, and autophagy induction [3].

**Figure 1:**
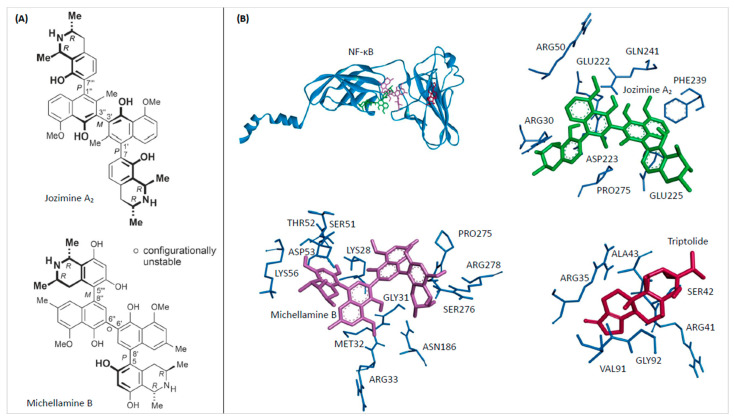
The (**A**) chemical structures of jozimine A_2_ and michellamine B and the (**B**) in silico molecular docking analysis. The docking poses and interacting amino acid residues of NF-κB (PDB ID: 1NFI) with michellamine B (violet), jozimine A_2_ (green), and the positive control triptolide (red) separately and all together. Each docking analysis was carried out three times.

Some of these structurally and biosynthetically unique secondary metabolites also occur as dimers, such as jozimine A_2_ (for the structure, see Figure 1A), which is the (twofold *O*-demethylated) dimer of dioncophylline A, with the two dioncophylline A moieties being connected by a chiral binaphthyl axis [4].

Michellamine B was isolated from *Ancistrocladus likoko* following a procedure described earlier [5], while jozimine A_2_ was obtained from *Ancistrocladus ileboensis* as reported in the literature [6]. The chromatographic and spectroscopic data of the two pure metabolites were in accordance with those reported previously [4,7]. Both compounds were fully soluble in DMSO.

In several cases, dimeric NIQs have proven enhanced activities as compared to their monomeric analogs in several anti-infective test systems (e.g., against HIV, *Plasmodium*) [1,2,4,6]. More recently, some of these dimers, such as jozimine A_2_, have demonstrated strong antitumoral activities against pancreatic and cervical cancer cells, making the testing of their antileukemic activity a rewarding task.

As different studies have shown, NF-κB modulates a plethora of cellular processes, including cell proliferation, angiogenesis, and apoptosis suppression, making it an important link in promoting inflammation, cancer proliferation, and drug resistance [8,9,10,11,12,13,14,15,16]. NF-κB is activated in an inflammatory milieu and regulates various pro-inflammatory cytokines (TNFα, IL-6, IL-1β) as well as STAT3, a transcription factor that activates proliferative and survival genes in cancer cells [10,17,18,19]. Additionally, by activating various apoptosis-suppressing genes, NF-κB prevents apoptosis and promotes cancer cell proliferation in solid tumors, leukemias, and lymphomas [11,20,21,22,23,24]. Interestingly, there is also a complex interplay between NF-κB and autophagy, another type of programmed cell death, as the regulation of both pathways is interdependent [25]. Both apoptosis and autophagy have become attractive targets in cancer treatment. However, while apoptosis activation is always desired in cancer treatment, autophagy has a much more complex role and can induce tumor suppression or tumor proliferation, depending on the tumor stage [25,26,27,28]. Due to increasing multidrug resistance in cancer, numerous studies are now using natural products to target NF-κB and induce apoptosis or autophagy, leading to cancer suppression [3,16,29,30,31,32,33]. Furthermore, many of these phytochemicals, such as curcumin, resveratrol, and lycopene, exert their anti-cancer activity not only by targeting NF-κB but also by controlling epigenetic modifications, which are key regulators of apoptosis and autophagy [30,32,33,34,35,36].

In this paper, we describe the anticancer potential of jozimine A_2_, a naphthylisoquinoline alkaloid, on drug-sensitive CCRF-CEM leukemia cells and provide valuable information regarding its mechanism of action and the difference in activity in comparison to a structurally related but different NIQ alkaloid dimer, namely michellamine B. By using bioinformatic tools, we uncover the binding site that leads to the inhibition of the NF-κB pathway in silico. In addition, Ingenuity Pathway Analysis (IPA)^TM^ highlighted the

NF-κB pathway as the main network affected after treatment with jozimine A_2_. We further explored the mechanism by which jozimine A_2_ killed the cells. Moreover, jozimine A_2_ hampered the translocation of NF-κB to the nucleus, which led to the downregulation of NF-κB-dependent genes. Here, we report for the first time the mechanism of action of the anti-leukemic activity of jozimine A_2_.

## 2. Results

### 2.1. In Silico Binding of Jozimine A_2_ and Michellamine B to NF-κB

Molecular docking analysis, an integral technique in computer-aided drug discovery, was utilized to elucidate the binding interactions of jozimine A_2_ and michellamine B (for the structures, see Figure 1A) with the NF-κB protein. The calculated binding free energies of −11.11 ± 0.02 kcal/mol and −8.20 ± 0.00 kcal/mol for michellamine B and jozimine A_2_, respectively, demonstrated favorable predicted protein-ligand affinities (Table 1). However, as shown in Figure 1B, the analysis of the precise molecular docking conformations revealed distinct NF-κB binding sites for both natural products compared to the reference inhibitor triptolide. While binding in close proximity to one another, the specific amino acid residues involved in the predicted NF-κB complexes significantly differed between michellamine B and jozimine A_2_.

Both molecules investigated in this study have three biaryl axes as characteristic structural features. Depending on the degree of steric hindrance, such single bonds between aromatic ring systems can freely rotate—or can be rotationally hindered—and can then be an element of axial chirality (besides the likewise present stereogenic centers).

In the case of jozimine A_2_, all three axes are rotationally hindered; they are P-, M-, and P-configured. Any docking approach has to consider that this axial configuration does not change during the docking process. Indeed, the structure of jozimine A_2_ in the binding pocket has still retained its P,M,P configuration: M at the central axis and P at the two outer ones.

On the contrary, in the related dimeric alkaloid michellamine B, only the two outer axes are rotationally stable: one is M-configured and one is P, while the central axis, having much less steric hindrance from neighboring ortho-substituents, can freely rotate at room temperature, thus achieving a fast M,P-interconversion. Any docking procedure must recognize the stable axial arrays for the outer axes while accepting free rotation for the inner one. In the pocket, michellamine B indeed shows the expected axial configurations at the outer axes, once M and once P. The central axis is found to be P in the energetically lowest conformation, but there are both P- and M-configured geometries among the energetically next-higher species, clearly proving the procedure to accept free rotation at the central axis, fully in agreement with the experimental reality.

The current work is an illustrative example of the successful docking of a natural product possessing both configurationally stable and unstable biaryl axes.

### 2.2. Jozimine A_2_ and Michellamine B Both Bind to NF-κB In Vitro, but Only Jozimine A_2_ Inhibits NF-κB Activity

**Figure 2:**
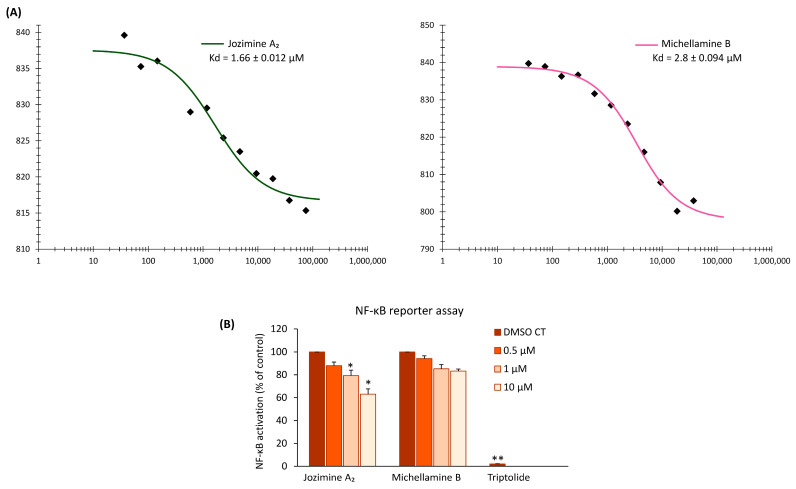
Microscale thermophoresis and NF-κB reporter assay for jozimine A_2_ and michellamine B. (**A**) Binding curve of jozimine A_2_ and michellamine B to NF-κB generated as a function of concentration-dependent changes in the fluorescence signal. The calculated dissociation constant (K_d_) is 2.8 ± 0.094 µM for michellamine B and 1.66 ± 0.012 µM for jozimine A_2_. (**B**) NF-κB activity after treatment with jozimine A_2_, michellamine B, and triptolide at different concentrations compared to DMSO control. * *p* < 0.05, ** *p* < 0.01.

Microscale thermophoresis (MST) was used to determine the affinity of jozimine A_2_ and michellamine B towards recombinant NF-κB proteins. This biophysical assay is often used to investigate the interactions between ligands and proteins [37] and represents a good way to confirm the in silico data. The MST analysis revealed that both jozimine A_2_ and michellamine B strongly bind to the NF-κB protein, with a dissociation constant (K_d_) of 2.8 ± 0.094 µM for michellamine B and 1.66 ± 0.012 µM for jozimine A_2_ (Figure 2A). After both in silico (molecular docking) and in vitro (MST) assays showed the potential jozimine A_2_ and michellamine B have on NF-κB, the next step was to determine whether these compounds can inhibit NF-κB translocation. For this purpose, the reporter cell line HEK-Blue^TM^ Null 1 was treated with increasing concentrations of the respective compounds. Interestingly, jozimine A_2_ significantly inhibited NF-κB activity in a dose-dependent manner by 40%, whereas michellamine B only weakly and not significantly decreased NF-κB activity in comparison to DMSO as the control (Figure 2B).

### 2.3. Cytotoxicity of Jozimine A_2_ and Michellamine B

**Figure 3:**
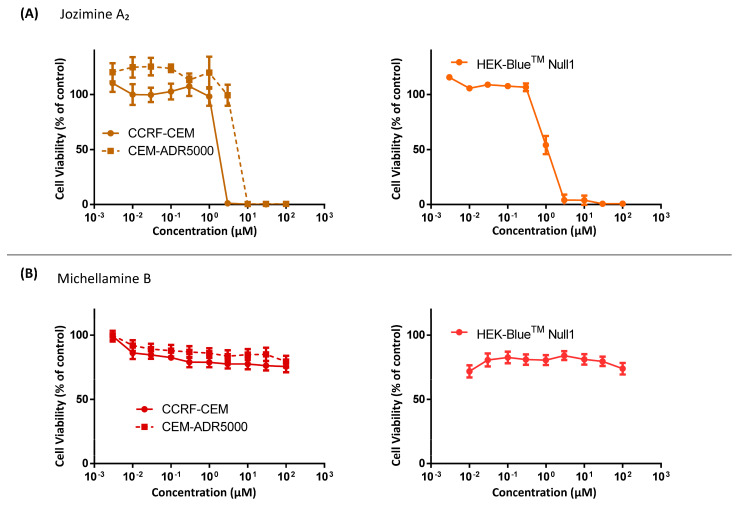
Cytotoxic effects of jozimine A_2_ and michellamine B by the resazurin reduction assay. Dose–response curves of jozimine A_2_ (**A**) and michellamine B (**B**) on CCRF-CEM and drug-resistant CEM-ADR5000 leukemia cell lines and on HEK-Blue^TM^ Null 1 cells. The data are the result of mean values ± SD of three independent experiments.

The cytotoxic activity of jozimine A_2_ and michellamine B was evaluated in human CCRF-CEM and multidrug-resistant CEM/ADR5000 leukemia cells utilizing a resazurin reduction assay. Figure 3A demonstrates that jozimine A_2_ had potent cytotoxicity towards both CCRF-CEM (IC_50_ = 0.45 ± 0.00 μM) and CEM/ADR5000 (IC_50_ = 0.69 ± 0.05 μM) cell lines. The IC_50_ values are shown in Table 2. Conversely, michellamine B (Figure 3B) did not prevent cell growth in either cell line. Additionally, jozimine A_2_ suppressed the viability of HEK-Blue^TM^ Null 1 cells with an IC_50_ of 0.54 ± 0.01 μM, affirming its broad cytotoxic efficacy across multiple human cell types. Because michellamine B did not show any cytotoxic effect, it was not further investigated.

### 2.4. Validation of Microarray Analysis Using qRT-PCR

**Figure 4:**
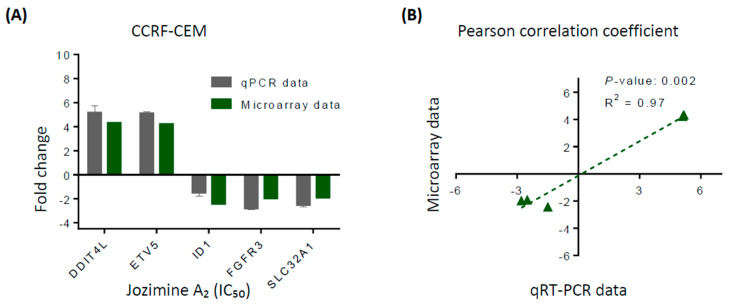
Validation of microarray hybridization using qPCR. (**A**) Technical verifications of the top up-and downregulated genes in CCRF-CEM cells after treatment with jozimine A_2_. Gene expression of *GAPDH* served as internal control. (**B**) Pearson correlation coefficient (*R*^2^ = 0.97) shows a high correlation between the microarray and the qRT-PCR data.

The top affected genes were used to confirm the microarray analyses by the qRT-PCR method. Treatment of CCRF-CEM cells with jozimine A_2_ with an IC_50_ concentration increased the expression of DDIT4L and ETV5 genes and decreased the expressions of ID1, FGFR3, and SLC32A1, as observed in the microarray analyses (Figure 4A). The correlation between the qPCR and microarray was calculated using the Pearson correlation test (r^2^ = 0.985) (Figure 4B).

### 2.5. The Effect of Jozimine A_2_ on the Cell Cycle

**Figure 5:**
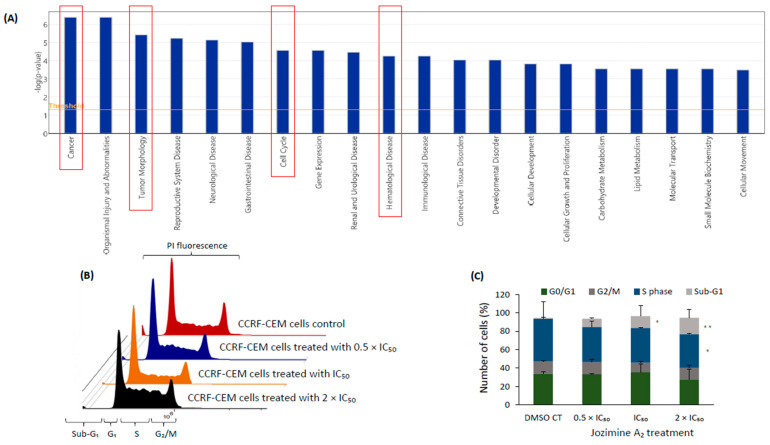
The effect of jozimine A_2_ on the cell cycle. (**A**) Canonical pathways identified by the Ingenuity Pathway Analysis (IPA) of CCRF-CEM cells following treatment with jozimine A_2_ for 24 h. (**B**) Histogram of cell cycle distribution and (**C**) percentage of cells in the different cell cycle phases upon treatment with different concentrations of jozimine A_2_ (0.5 × IC_50_, IC_50_, 2 × IC_50_) for 24 h. A significant accumulation can be observed in the sub-G_1_ phase, indicating the presence of apoptotic cells. * *p* < 0.05, ** *p* < 0.01.

The IPA analysis predicted that one of the major canonical pathways affected after treatment with jozimine A_2_ was the cell cycle, in addition to other diseases and functions such as tumor morphology, hematological diseases, and cancer (Figure 5A). Jozimine A_2_ induced the accumulation of cells in the sub-G_1_ phase in a dose-dependent manner. Already at a concentration of 0.5 × IC_50_, the sub-G_1_ phase increased to 8.94%, going to 12.93% at IC_50_ and reaching 17.85% at 2 × IC_50_. The cell population in the G_0_/G_1_ phase was 33.4% for 0.5 × IC_50_ and 35.55% for IC_50_ and decreased to 27.25% at 2 × IC_50_ concentration (Figure 5B,C).

### 2.6. Jozimine A_2_ Prevents NF-κB Translocation and Suppresses the Expression of NF-κB-Dependent Genes

**Figure 6:**
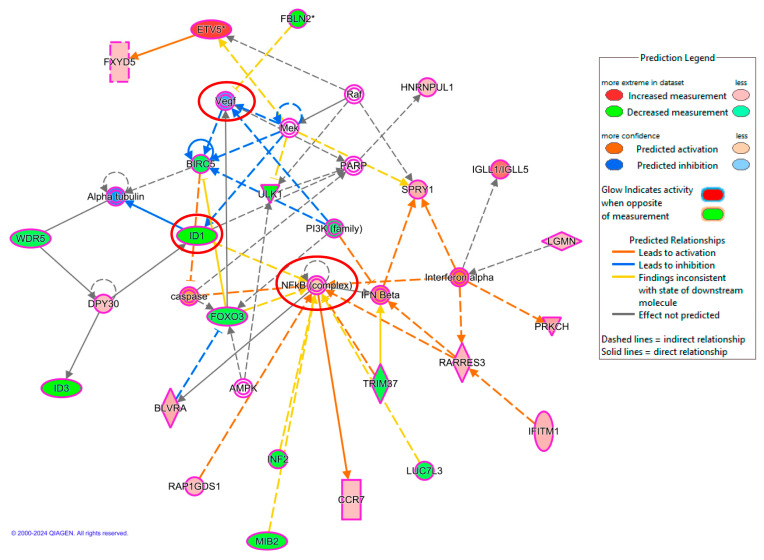
Microarray analysis using IPA, indicating that NF-κB network is affected upon treatment with jozimine A_2_. NF-κB-related genes *ID1* and *VEGF* are downregulated. The colors green and blue indicate inhibition/downregulation, while red means activation/upregulation. Duplicates Gene/Protein/Chemical identifiers marked with an asterisk indicate that multiple identifiers in the dataset file map to a single gene/chemical in the Global Molecular Network.

**Figure 7:**
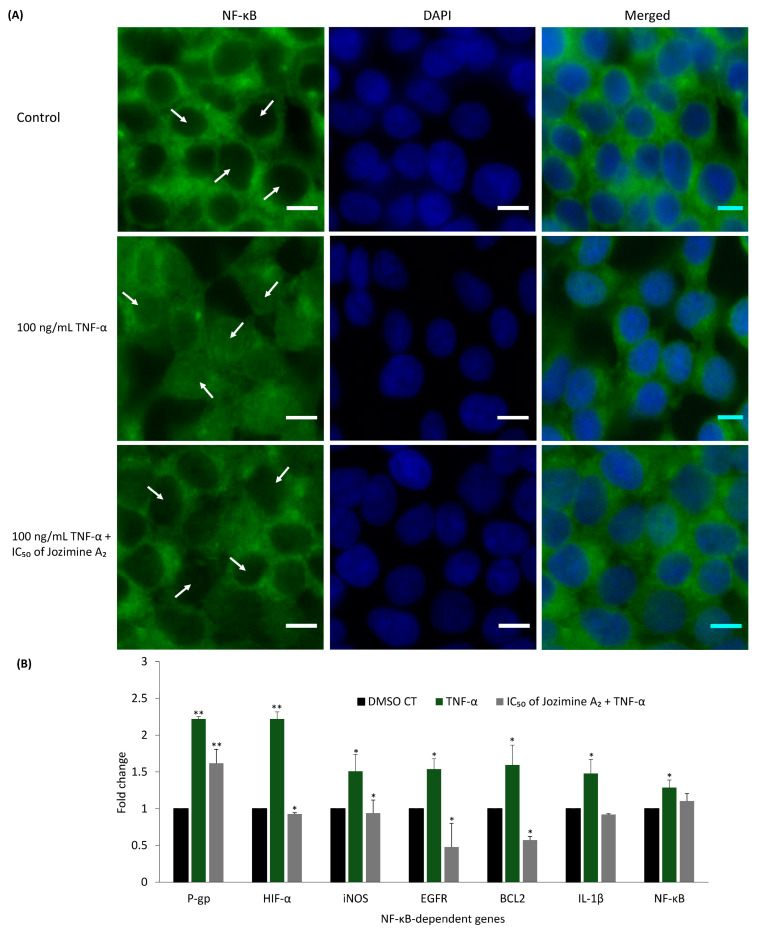
(**A**) Visualization of NF-κB translocation in HEK-Blue^TM^ Null1 cells. Treatment with TNF-α indicates the translocation of NF-κB to the nucleus, while incubation with jozimine A_2_ for 24 h prevents the translocation. The data were acquired using a AF7000 widefield fluorescence microscope at 40× magnification (scale bars = 10 μm). (**B**) Fold change in the expression of NF-κB-dependent genes upon treatment with DMSO, TNF-α and IC_50_ jozimine A_2_ + TNF-α. The gene expression was measured using qPCR with *GAPDH* as internal control. * *p* < 0.05, ** *p* < 0.01. Treatment with TNF-α indicates the translocation of NF-κB to the nucleus, while incubation with jozimine A2 for 24 h prevents the translocation.

The IPA analysis showed that NF-κB is one of the networks most strongly affected by jozimine A_2_, with downregulated expression of genes related to tumor progression, such as ID1 and VEGF (Figure 6). Immunofluorescence was used to verify whether jozimine A_2_ could prevent NF-κB translocation to the nucleus following TNF-α activation. In DMSO-treated control cells, NF-κB was translocated to the nucleus following treatment with TNF-α (Figure 7A). However, incubation with jozimine A_2_ prevented the translocation despite the addition of TNF-α (Figure 7A). In addition to the immunofluorescence experiments, the qPCR results indicated that treatment with TNF-α increased the expression of NF-κB-dependent genes, such as HIF-α, iNOS, and IL-1β, compared to DMSO as the control, while treatment with TNF-α and jozimine A_2_ significantly decreased the expression of these genes (Figure 7B). Thus, jozimine A_2_ modulates the NF-κB pathway.

### 2.7. Suppression of Endothelial Tube Formation by Jozimine A_2_

**Figure 8:**
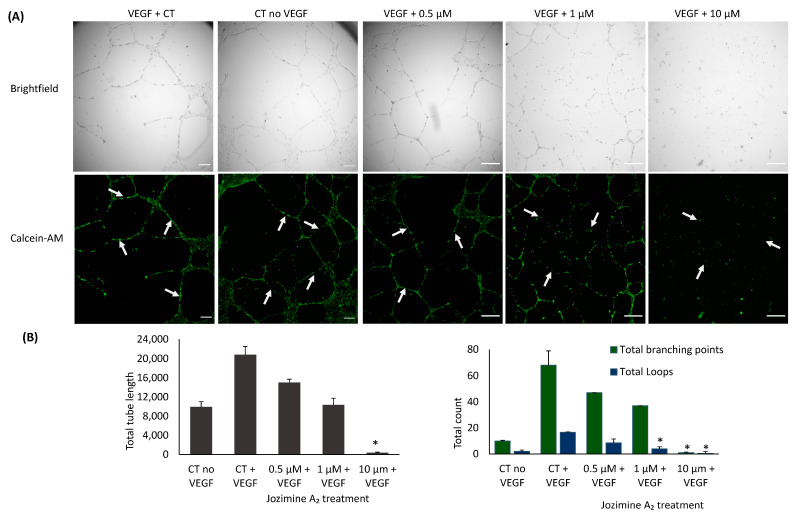
Inhibition of endothelial tube formation by jozimine A_2_. (**A**) Images of cells after treatment with three different concentrations of jozimine A_2_ and VEGF, following the method we have previously described. This experiment was performed at the same time together with other compounds in the same set of experiments; therefore. Arrows point to the tube areas. (**B**) Jozimine A2 suppresses angiogenesis in a dose-dependent manner. This is indicated by a reduction in the total tube length and total branching points in HUVECs. * *p* < 0.05.

Jozimine A_2_ was also investigated for its potential to prevent endothelial tube formation. HUVECs were incubated with increasing concentrations (0.5 µM, 1 µM, and 10 µM) of jozimine A_2_. As Figure 8 indicates, jozimine A_2_ dose-dependently suppressed endothelial tube formation by inhibiting both total branching points and tube length after VEGF induction. Angiogenesis suppression had also been predicted by IPA (Figure 6), as the same genes regulating NF-κB signaling are also related to angiogenesis, namely ID1 and VEGF, which were downregulated (Figure 6).

### 2.8. Jozimine A_2_ Induces Cell Death through Apoptosis by Autophagy

**Figure 9:**
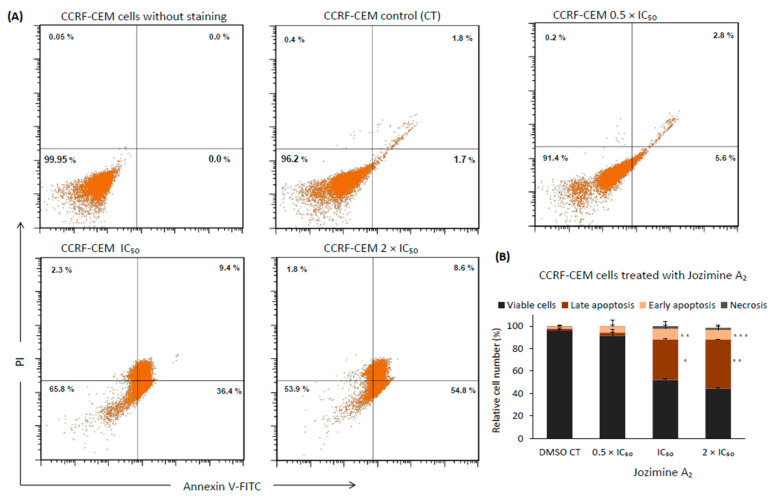
Apoptosis detection using flow cytometry. (**A**) Apoptosis was monitored using annexin V/PI staining. Percentage of late apoptosis increased from 1.7% in control samples to 54.8% in 2 × IC_50_. (**B**) Treatment with 0.5 × IC_50_, IC_50_, 2 × IC_50_ for 48 h increased the percentage of early and late apoptotic cells and necrotic cells. The data are the result of mean values ± SD of three independent experiments. * *p* < 0.05, ** *p* < 0.01, *** *p* < 0.001.

As the results from the cell cycle indicated an accumulation of apoptotic cells (sub-G_1_ phase), it seemed rewarding to understand how jozimine A_2_ induces cell death. As such, two different cell death mechanisms were investigated: apoptosis and autophagy. Jozimine A_2_ treatment dose-dependently triggered apoptotic cell death in CCRF-CEM cells after treatment for 48 h. Exposure to jozimine A_2_ induces early apoptosis already at 0.5 × IC_50_ (Figure 9A,B). Increasing levels of late apoptotic cells were measured at higher concentrations, IC_50_ (36.4%) and 2 × IC_50_ (54.8%), compared to the DMSO-treated control (1.7%) (Figure 9A,B). At higher concentrations, necrotic cell fractions appeared, namely 2.34% for IC_50_ and 1.8% at 2 × IC_50_, compared to the DMSO-treated control (0.4%) (Figure 9A,B).

**Figure 10:**
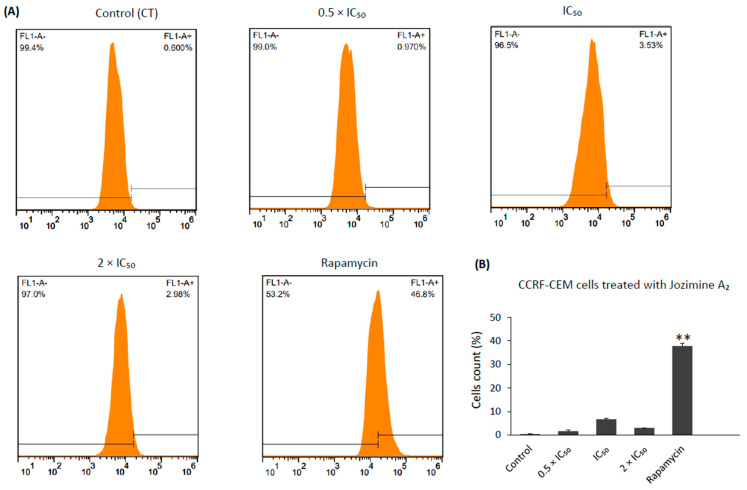
Stimulation of autophagy in CCRF-CEM cells by jozimine A_2_. (**A**) Histograms obtained from flow cytometry showing the induction of autophagy by jozimine A_2_ treatment (0.5 × IC_50_, IC_50_, and 2 × IC_50_) and rapamycin (1 μM). (**B**) Bar chart illustrating the significant increase in autophagy by jozimine A_2_ intervention for 24 h in a dose-dependent manner from two independent experiments. ** *p* < 0.01.

On the other hand, autophagy was induced to a lesser extent. Jozimine A_2_ exerted autophagy at IC_50_ and 2 × IC_50_ to a percentage of 6.8 and 3.2 of the autophagic cells, respectively, compared to the untreated control from two independent experiments. However, treatment with rapamycin, which was used as a positive control, induced 37.8% of the autophagic cells (Figure 10). Thus, jozimine A_2_ preferentially induces apoptosis as the main cell death mechanism.

## 3. Discussion

Jozimine A_2_ and michellamine B are bioactive dimeric naphthylisoquinoline (NIQ) alkaloids produced by tropical *Ancistrocladus* lianas, with marked anti-plasmodial and anti-HIV activities, respectively [4,7]. The NIQ alkaloids are an emerging class of active secondary metabolites with increased pharmaceutical potential, as members of this class, depending on their individual structure, are active against various pathogens causing tropical diseases and against different cancer cells [4,5,38]. Additionally, jozimine A_2_ also showed increased anticancer activity towards fibrosarcoma (HT1080), multiple myeloma (MM.1S), human colon carcinoma (HT-29), and human pancreatic cancer (PANC-1) cells, making it a promising therapeutical candidate in the battle against cancer [38].

Recently, our group described how dioncophylline A, a related but monomeric NIQ alkaloid, can induce autophagy, leading to cell death in acute leukemia cells, and act as an NF-κB inhibitor [3]. Therefore, it was interesting to investigate whether the two NIQ alkaloid dimers, jozimine A_2_ and michellamine B, have similar activities as the monomeric dioncophylline A.

In the molecular docking experiments, both dimers showed high binding energies, particularly michellamine B (−11.1 kcal/mol), which was significantly stronger than for jozimine A_2_, with its still very good energy (−8.2 kcal/mol) towards NF-κB. This stronger binding for michellamine B is certainly due to the higher number of free OH groups, which may give rise to additional interactions and a higher polarity. Also advantageous for the high affinity of michellamine B might be the better flexibility of the molecule, which can thus more easily adapt its conformation to the geometry inside the pocket.

Despite all the similarities, there are some obvious differences between the two alkaloids. Firstly, concerning the functional groups: Jozimine A_2_ has two fewer free OH groups than michellamine B; thus it is more lipophilic. Secondly, regarding their molecular shape: because of the position of the outer axes of jozimine A_2_, the molecule is slimmer than the more compact michellamine B, which can influence its docking behavior. Lastly, from a stereochemical point of view, in jozimine A_2_, the two outer axes are both *P*-configured, while in michellamine B, one outer axis is *M* and one is *P*. The central axis in jozimine A_2_ is rotationally hindered, too, making it more rigid, while in michellamine B, the central axis is flexible, which makes it easier for the molecule to slip into a pocket in a docking process.

The promising results on NF-κB obtained from molecular docking and MST encouraged us to verify in vitro if the two dimers can prevent NF-κB translocation to the nucleus and check the cytotoxic potential of these new compounds on leukemia cells. Surprisingly, the in vitro behavior of the two alkaloids was found to be different, as the NF-κB reporter assay shows: Jozimine A_2_ significantly blocks NF-κB activity compared to michellamine B, which only displays a weak effect on NF-κB. Furthermore, the cytotoxicity assay revealed that jozimine A_2_ prevents cell proliferation in drug-sensitive cells (CCRF-CEM) with an excellent IC_50_ = 0.45 ± 0.00 μM, comparable to the standard doxorubicin, which has an IC_50_ = 0.14 μM, while the IC_50_ of michellamine B was not reached (>100 µM) [3]. Moreover, jozimine A_2_ also inhibited cell proliferation of multidrug-resistant CEM/ADR5000 leukemia cells at IC_50_ = 0.69 ± 0.05 μM. The higher flexibility, the larger polarity (and better water solubility), and the more compact structure do not seem to play a significant role in vitro. The higher lipophilicity of jozimine A_2_ should entail better membrane permeability and, thus, a higher availability of the agent in the cells. In addition to the NF-κB reporter assay, the immunofluorescence highlighted that jozimine A_2_ modulated the NF-κB pathway, not only by suppressing its activity but also by blocking its translocation to the nucleus. Given the necessity for new chemotherapeutic agents due to the increasing number of drug-resistant tumors, jozimine A_2_ has proven to be a valuable chemotherapeutic candidate [39,40]. Therefore, it was selected for further investigation, to decipher its underlying mechanism.

The IPA analysis identified “cancer”, “cell cycle”, “hematological diseases”, and “tumor morphology” among the top canonical pathways affected by the treatment with jozimine A_2_. According to the microarray analysis, the top deregulated genes after treatment with jozimine A_2_ were *DDIT4L*, *ETV5*, *ID1*, *FGFR3*, and *SLC32A1*. These results were confirmed using qPCR. *DDIT4L* is a p53-dependent DNA damage repair gene and an mTOR inhibitor, which has been identified as a potential biomarker in several cancers [41]. *SLC32A1* is aberrantly methylated in pancreatic cancer and has been identified as an NF-κB-inducible gene [42,43]. *FGFR3* expression has been correlated with tumor growth in different cancer types and is a known regulator of the NF-κB pathway [44,45]. Additionally, jozimine A_2_ does not only prevent translocation of NF-κB to the nucleus, but it also blocks tumor-progression genes such as *ID1* and *VEGF.*

Given the positive results obtained, we decided to further investigate the mechanisms behind the anti-tumor activity of jozimine A_2_. Angiogenesis is an important factor in tumor growth and metastasis, and numerous drugs have been developed to inhibit angiogenesis [3,46,47]. Therefore, the next step was to investigate how jozimine A_2_ influences endothelial tube formation in vitro. As the microarray and qPCR analyses showed a downregulation of *ID1* and *VEGF*, it was not surprising that jozimine A_2_ reduced endothelial tube formation and, consequently, angiogenesis. This is in accordance with our previous study, which described the potential of monomeric NIQ alkaloids to inhibit angiogenesis [3].

Although strong cytotoxic activities against various cancer types have been described for several NIQ alkaloids [3,26,27,28,29,41,42,43], the triggering mechanisms have not yet been deciphered. As this unique pharmacological class of natural products is emerging, there is still much to learn about the mechanisms of action of each individual structure. Given the results from the IPA analysis regarding the “cell cycle,” we decided to investigate the impact of jozimine A_2_ on the cell cycle. As the results indicated, jozimine A_2_ does not induce cell cycle arrest. However, in view of the observed accumulation of cell debris (sub-G_1_ phase), it seemed rewarding to investigate both apoptosis and autophagy as possible modes to induce cell death. Several studies have described apoptosis or autophagy as the main induced cell death mode in different cancer cells [3,5,27,28,29,48,49,50,51,52]. Our investigations show that jozimine A_2_ preferentially dose-dependently induces apoptosis in CCRF-CEM cells, as the number of late-apoptotic cells was significantly increased compared to the control. In comparison, autophagy was only weakly induced by 6% after treatment with jozimine A_2_, which was much lower than the positive control, rapamycin, which induced autophagy by 37%.

## 4. Materials and Methods

### 4.1. Molecular Docking

Jozimine A2 and michellamine B were obtained by isolation from Central African Ancistrocladus lianas [5,6]. The structures of jozimine A_2_ and michellamine B were formed manually on ChemDraw 19.0, https://perkinelmerinformatics.com/products/research/chemdraw, Massachusetts, USA (accessed on 1 February 2021), by using the template of the sdf structure of dioncophylline A 3D, which was downloaded from the PubChem database, https://pubchem.ncbi.nlm.nih.gov/compound/Dioncophylline-A Maryland, USA (accessed on 1 August 2022 ). After energy minimization, the compounds were saved as pdb files on Chem3D 19.0, https://perkinelmerinformatics.com/products/research/chemdraw, Massachusetts, USA (accessed on 1 February 2021), and converted to pdbqt files by PyRx. Triptolide as the positive control was also downloaded from PubChem and went through the same procedure. For jozimine A_2_, all three axes were rotationally hindered, while for michellamine B, the central axis was left freely rotating, and two outer axes were hindered on AutoDockTools 1.5.6., https://ccsb.scripps.edu/mgltools/, California, USA (accessed on 24 January 2021). The stereostructures of the two compounds were confirmed by one of the authors (G.B.).

The NF-κB p65/p50 heterodimer bound to the IκBα protein complex was obtained from the Protein Data Bank (PDB ID 1NFI) as a PDB file. The inhibitor IκBα chain, p50 chain, and heteroatoms were removed to isolate the p65 subunit. Polar hydrogens and Kollman charges were added to the p65 protein structure, which was then converted to a PDBQT file using AutoDockTools. A grid box centered at x = 3.348, y = 44.715, z = 17.684 with dimensions of 80 × 70 × 126 points and 0.4 Å spacing was generated around the protein. The gpf, glf, and dpf affinity map files were also prepared. Molecular docking using a Lamarckian algorithm was performed utilizing the supercomputing facilities at Johannes Gutenberg University Mainz, hpc.uni-mainz.de, Mainz, Germany (accessed on 30 August 2022). Docking runs totaling 250 iterations with a maximum of 2,500,000 energy evaluations were completed for each compound assessed.

### 4.2. Microscale Thermophoresis

Microscale thermophoresis (MST) was used to confirm the docking results. The human recombinant NF-κB protein was purchased from Sino Biological Europe GmbH (NF-κB cat. No. 12054-H09E, Sino Biological Europe GmbH, Eschborn, Germany), and the labeling was performed as previously described [53]. Serial dilutions from 300,000 to 100 nM of jozimine A_2_ and michellamine B were incubated for 30 min at room temperature (RT) with the labeled human recombinant NF-κB protein in a 1:1 ratio. Standard capillaries from NanoTemper Monolith™ NT (NanoTemper Technologies GmbH, Munich, Germany) were used to conduct the experiments. MST was run at 40% LED power and 10% MST power for jozimine A_2_ and 40% LED power and 40% MST power for michellamine B.

### 4.3. NF-κB Reporter Assay

1 × 10^5^ NF-κB SEAP reporter HEK-Blue^TM^ Null 1 cells, acquired from InvivoGen (San Diego, CA, USA), were seeded on a 96-well plate and incubated for 24 h. Afterwards, the cells were treated with increasing concentrations (0.5 µM, 1 µM, and 10 µM) of jozimine A_2_ and michellamine B, respectively, for another 24 h. Tumor necrosis factor (TNF, 100 ng/mL, Thermofisher; Darmstadt, Germany) was added to activate the NF-κB pathway. Triptolide (1 µM, InvivoGen, San Diego, CA, USA) served as a positive control [52]. After adding pre-heated Quanti-Blue (InvivoGen, San Diego, CA, USA), the fluorescence signal was measured on an Infinite M2000 Tecan (Tecan, Crailsheim, Germany) at 630 nm. The experiment was performed in triplicate.

### 4.4. Cytotoxicity Assay

The cytotoxic effects of jozimine A_2_ and michellamine B were assessed using a resazurin reduction assay (Promega, Mannheim, Germany) and DMSO (dimethylsulfoxide) as a negative control. In this assay, living cells reduce non-fluorescent resazurin dye to fluorescent resorufin [54].

CCRF-CEM cells and multidrug-resistant CEM/ADR5000 cells were kindly provided by Prof. Axel Sauerbrey (Department of Pediatrics, University of Jena, Germany). The multidrug resistance phenotype of CEM/ADR5000 cells with resistance to anthracyclines, *Vinca* alkaloids, taxanes, and epipodophyllotoxines has been reported [55]. HEK-Blue^TM^ Null 1 cells were purchased commercially (InvivoGen, San Diego, CA, USA).

All cells seeded in 96-well culture plates were treated with varying concentrations (0.003 µM to 100 µM) of the two compounds for 72 h (37 °C). HEK-Blue^TM^ Null 1 cells were pre-incubated for 24 h to allow attachment prior to treatment. After treatment, 20 μL of 0.01% resazurin solution (Sigma-Aldrich, Schnelldorf, Germany) were added to each well and incubated for another 4 h (37 °C). Fluorescence was measured at excitation 544 nm/emission 590 nm on an Infinite M2000 Pro plate reader (Tecan, Crailsheim, Germany). Cell viability percentages were calculated by comparing the fluorescence of treated vs. untreated cells with a medium background subtracted. IC_50_ values were determined using nonlinear regression analysis relative to DMSO as the control. The assay was performed in triplicate with six replicate wells per concentration. IC_50_ values are expressed as the mean ± standard deviation.

### 4.5. Microarray Hybridization

Microarray hybridization expression analysis was carried out to identify the underlying mechanisms of jozimine A_2_ anticancer activity. Therefore, CCRF-CRM cells were incubated for 24 h with jozimine A_2_ at the IC_50_ concentration. The InviTrap^®^ Spin Universal RNA Mini Kit (Invitek Molecular, Berlin, Germany) was used for the extraction of the total RNA using the protocol provided by the manufacturer. Microarray hybridization for both treated and untreated samples was conducted on Illumina Human HT-12 BeadChip arrays (Illumina, San Diego, CA, USA) at the Genomics and Proteomics Core Facility at the German Cancer Research Center (DKFZ, Heidelberg, Germany). Data were analyzed through the Chipster software (https://chipster.csc.fi), and deregulated genes were then further investigated using the Ingenuity Pathway Analysis (IPA) software (QIAGEN® Ingenuity Pathway Analysis Fall Release (2023)) as previously described [3,56].

### 4.6. Quantitative Real-Time PCR

After RNA was isolated by the above-described method, 1 µg of RNA was converted to complementary DNA (cDNA) using the Luna Script™ RT SuperMix Kit (E3010, New England Bio Labs, Darmstadt, Germany). To amplify the genes as per the manufacturer’s instructions, 5 × Hot Start Taq EvaGreen^®^ qPCR Mix (no ROX) (Axon Labortechnik, Kaiserslautern, Germany) was used next. The NCBI/Primer-BLAST tool was used to design the primers, which were then purchased from Eurofins Genomics (Ebersberg, Germany). A list with the primers name and sequences can be found in Table 3. The sequence of the primers is the same that we used in our previously published work [3,57]. To conduct the qPCR in 40 cycles on CFX384™ (Bio-Rad, Munich, Germany), 384 well-plates were used. The running conditions were as follows: denaturation at 95 °C for 15 s, gradient annealing temperatures at 62–47 °C for 30 s, and a 1 min elongation step at 72 °C. The Cq values were calculated by Bio-Rad CFX Manager Software. The gene expression was then normalized using *GAPDH* as the control, and the fold change was calculated by applying the 2^−ΔΔCt^ method [58].

### 4.7. Cell Cycle

CCRF-CEM cells (1 × 10^6^ cells/well) were seeded in a 6-well plate and treated with different concentrations (0.5 × IC_50_, IC_50_, 2 × IC_50_) of jozimine A_2_ or DMSO for 24 h. The cells were centrifuged at 350× *g* for 5 min at 10 °C and washed with ice-cold PBS. To fix the cell pellet, ice-cold ethanol 75% was added and the cells were kept at −20 °C for 3 h. The cells were then washed again with PBS and 5 µg RNAse A (Merck KgaA, Darmstadt, Germany) was added to the samples. Propidium iodide in PBS (PI, 50 μg/mL) (Merck KgaA, Darmstadt, Germany) was used for cell staining. The measurements were carried out in a BD Accuri^TM^ C6 Flow Cytometer (BD Biosciences, Becton Drive, Franklin Lakes, NJ, USA) [3,59].

### 4.8. Immunofluorescence

After seeding in chamber slides (Nune), HEK-Blue^TM^ Null 1 cells were incubated with jozimine A_2_ at 0.5 × IC_50_ concentration and triptolide, respectively, for 24 h. NF-κB activation was induced by adding 100 ng/mol TNF after treatment for 24 h. The cells were washed three times with PBS and fixed with 4% paraformaldehyde for 30 min at room temperature. The monolayer was then blocked for 1 h in 3% bovine serum albumin and Triton-X 100. After washing, the diluted anti-NF-κB antibody (1:200, D2E5, Cell Signaling, Frankfurt am Main, Germany) was added and incubated overnight at 4 °C. The slides were washed three times and incubated with the secondary antibody Alexa Fluor 488-conjugated anti-rabbit (1:700, Invitrogen, A11032, Darmstadt, Germany) for 1 h. Lastly, 2 μg/mL 4′,6-diamidino-2-phenylindole (DAPI) (Sigma-Aldrich) was added for staining, and the cells were mounted in Fluoromount-G^®^ (SouthernBiotech, Birmingham, AL, USA) [3]. The images were acquired using an AF7000 Widefield Fluorescence Microscope (40 × magnification).

### 4.9. Endothelial Tube Formation

The tube formation assay was used to examine the potential of jozimine A_2_ for angiogenesis. Μ-Slide angiogenesis (ibidi GmbH, Martinsried, Germany) was coated with 10 μL Matrigel^®^ (Corning GmbH, Kaiserslautern, Germany) and polymerized for 30 min. Next, human umbilical vein endothelial cells (HUVECs) (1 × 10^4^ cells/well), received from Dr. Ronald E. Unger (Repair-Lab, Institute of Pathology, University Medical Center of Johannes Gutenberg University Mainz, Mainz, Germany), were seeded on Matrigel^®^ with 30 ng/mL VEGF (Promocell, Heidelberg, Germany). For the tube formation, jozimine A_2_ (0.5 µM, 1 µM, and 10 µM) or DMSO (control) was added to the cells and incubated for 16–18 h at 37 °C and 5% CO_2_. As a next step, the cells were stained with 6.25 μg/mL Calcein-AM (Cayman Chemical, Ann Arbor, MI, USA) for 30 min at RT and then rinsed three times with Hank’s balanced salt solution (HBSS) (Corning B. V. Life Sciences, Amsterdam, Niederland). The fluorescent images were obtained with the F7000 widefield fluorescence microscope (Microscopy & Histology Core Facility at IMB Mainz, Mainz, Germany). The tube analysis was carried out using Wimasis’ Image Analysis Platform (https://mywim.wimasis.com/ accessed on 1 March 2023) [60].

### 4.10. Apoptosis

Apoptosis was detected using the Annexin V-FITC/PI double-staining kit (Invitrogen, Life Technologies GmbH, Darmstadt, Germany) by flow cytometry. In a 6-well plate, CCRF-CEM cells (1 × 10^6^ cells/well) were seeded for 24 h. The cells were treated with increasing concentrations (0.5 × IC_50_, IC_50_, 2 × IC_50_) of jozimine A_2_ or DMSO (control) for 24 h and 48 h. As a next step, the cells were harvested and washed once with cold PBS and 1 × annexin binding buffer. After centrifugation, the cells were stained by adding 5 µL of Annexin V-FITC and propidium iodide (PI) and incubated at RT in the dark for 15 min. Apoptosis was detected by flow cytometry (BD Accuri^TM^ C6, BD Biosciences, Becton Drive, Franklin Lakes, NJ, USA). The experiments were performed in triplicate.

### 4.11. Autophagy

The Autophagy Detection Kit (ab139484, Abcam, Cambridge, UK) was used to detect autophagy according to the manufacturer’s instructions. CCRF-CEM cells were exposed to various concentrations of jozimine A_2_ (0.5 × IC_50_, IC_50_, 2 × IC_50_) and rapamycin (0.5 µM) as a positive control for 24 h. Cells were washed twice using the 1 × assay buffer provided in the Autophagy Detection Kit and were then centrifuged. The cell pellets were resuspended in 250 µL cell culture medium supplemented with 5% FBS and without indicators. 250 µL medium containing green stain solution (from the Detection Kit) was added, and cells were incubated for 30 min at 37 °C. Finally, the cells were washed again and re-immersed in the assay buffer. The autophagy signal was detected on the BD Accuri^TM^ C6 flow cytometer (BD Biosciences, Becton Drive, Franklin Lakes, USA) using a standard green (FL1) filter [3,59].

## 5. Conclusions

In conclusion, jozimine A_2_, a dimeric natural NIQ alkaloid, is a potential antitumor drug candidate through its cytotoxicity and NF-κB inhibition. Jozimine A_2_ bound to NF-κB both in silico and in vitro and inhibited its activity and translocation to the nucleus. Furthermore, jozimine A_2_ induced apoptosis in leukemia cells, as predicted by the IPA analysis and cell cycle investigation, and blocked angiogenesis by preventing endothelial tube formation.

## Figures and Tables

**Table 1:** Binding energies and predicted inhibition constants (pKi) are represented as mean ± SD.

Compound	Mean Binding Energy (kcal/mol)	pKi (µM)	Interacting Residues
Michellamine B	−11.11 ± 0.02	0.007 ± 0.000	LYS28, GLY31, MET32, ARG33, SER51, THR52, ASP53, LYS56, ASN186, PRO275, SER276, ARG278
Jozimine A2	−8.20 ± 0.00	0.979 ± 0.005	ARG30, ARG50, GLU222, ASP223, GLU225, PHE239, GLN241, PRO275
Triptolide	−6.15 ± 0.00	31.130 ± 0.070	GLY32, ARG35, ARG41, SER42, ALA43, VAL91

**Table 2:** Cytotoxic effects of jozimine A_2_ and michellamine B towards leukemia cell lines.

Cell Lines	IC_50_ of Jozimine A_2_ (µM)	IC_50_ of Michellamine B (µM)
CCRF-CEM	0.44 ± 0.001	>100
CEM/ADR5000	0.69 ± 0.04	>100
HEK-Blue^TM^ Null1	1.09 ± 0.003	>100

**Table 3:** Primer sequences of deregulated genes for qRT-PCR.

Gene	Forward Primer (5′ ⇒ 3′)	Reverse Primer (5′ ⇒ 3′)
DDIT4L	TCCTGAACCCAACCTCAACG	AAAGCCGCAGGACATCTTGA
ETV5	AGCTGTCTCTGGATTGGCAC	TTTGGTGGTTTTCTGCCCCT
ID1	GTGCCTAAGGAGCCTGGAAA	CCGCCTGTGAAAACGAGAAG
FGFR3	GGTGGGCTTCTTCCTGTTCA	GGGACACCTGTCGCTTGAG
SLC32A1	CGGCAGCTCCGCAGT	GCGTTCGAGGCTCTCTCAG
Pgp	CTGGTGTTTGGAGAA	GCCAGTGAAAAATGTTGCCAT
HIF1A	GTCTGAGGGGACAGGAGGAT	CTCCTCAGGTGGCTTGTCAG
I NOS	GCCATAGAGATGGCCTGTCC	GTGTCACTGGACTGGAGGTG
EGFR	GGTGAGTGGCTTGTCTGGAA	GTTTCCCCCTCTGGAGATGC
BCL2	GGATAACGGAGGCTGGGATG	TGACTTCACTTGTGGCCCAG
IL-1B	TCGCCAGTGAAATGATGGCT	GGTCGGAGATTCGTAGCTGG
NF-ĸB	CAATCACGATCGTCACCGGA	GGTCCGCTGAAAGGACTCTT

## Data Availability

Data are contained within the article.
